# Estimating the transfer function of cortical neurons : from simple models to in vitro experiments

**DOI:** 10.1186/1471-2202-15-S1-P173

**Published:** 2014-07-21

**Authors:** Yann Zerlaut, Gilles Ouanounou, Bartosz Teleńczuk, Charlotte Deleuze, Thierry Bal, Alain Destexhe

**Affiliations:** 1 Unité de Neurosciences, Information and Complexité, CNRS UPR 3293, Gif-sur-Yvette 91198, France

## 

The transfer function of the neuron maps its output firing rate to its synaptic inputs, and is a central function used in many computational models, such as typically mean-field models. To design realistic mean-field models, one must estimate the transfer function from real neurons, but for doing this, one should scan the whole synaptic input space, which is experimentally extremely challenging. One way to solve this problem is to obtain an analytic template that can fit the transfer function of complex neuron models, and use this template to guide the experiments. This is the approach that we have followed in this study. We introduce an analytic template and a fitting procedure that is able to capture the firing rate response of different neuronal models. The procedure was successful for models of increasing complexity from conductance-based Integrate-and-Fire to Hodgkin-Huxley models with adaptation (models of “regular-spiking” cells), which suggests that it may also work for real neurons. Based on this approach, we have performed experiments to calculate the transfer function from cortical neurons recorded in mice cortical slices. We developed a protocol based on the perforated patch technique that allows long recordings (~1-2h) with stable electrophysiological and excitability properties, as measured from the stability of response to the same protocol during the whole experiment. We used the dynamic-clamp setup to inject different combinations conductance inputs generated by excitatory and inhibitory shot-noises. The combination between the high number of sampling points (due to the recording technique) and the analytical template allowed us to have a very fine description of the neuronal transfer functions of layer 5 excitatory and inhibitory neurons in V1. This approach opens the path towards building biophysically-realistic mean-field models of cortical network activity.

